# Increased Community-Associated Infections Caused by Panton-Valentine Leukocidin–Negative MRSA, Shanghai, 2005–2014

**DOI:** 10.3201/eid2211.160587

**Published:** 2016-11

**Authors:** Min Li, Yanan Wang, Yuanjun Zhu, Yingxin Dai, Xufen Hong, Qian Liu, Tianming Li, Juanxiu Qin, Xiaowei Ma, Huiying Lu, Jie Xu, Michael Otto

**Affiliations:** Shanghai Jiao Tong University, Shanghai, China (M. Li, Y. Wang, Y. Zhu, Y. Dai, X. Hong, Q. Liu, T. Li, J. Qin, X. Ma, H. Lu, J. Xu);; National Institutes of Health National Institute of Allergy and Infectious Diseases, Bethesda, Maryland, USA (M. Otto)

**Keywords:** methicillin-resistant *Staphylococcus aureus*, MRSA, community-acquired infections, community-associated infections, Panton-Valentine leukocidin, PVL, bacteria, Shanghai, China

## Abstract

During 2005–2014, community-associated methicillin-resistant *Staphylococcus aureus* infections increased in Shanghai, China. Most infections were caused by sequence type 59 *S. aureus* that lacked Panton-Valentine leukocidin. This finding challenges the notion that Panton-Valentine leukocidin is necessary for epidemiologic success of community-associated methicillin-resistant *S. aureus*.

In the United States, community-associated (CA) methicillin-resistant *Staphylococcus aureus* (MRSA) infections in otherwise healthy persons in the community, first reported in the late 1990s (*1*), have reached epidemic dimensions (*2*). Despite considerable research efforts, the molecular underpinnings of the epidemiologic success of CA-MRSA are still not completely understood. Most typically connected with CA-MRSA is Panton-Valentine leukocidin (PVL). However, the role of PVL in CA-MRSA infection is controversial, primarily because of contradictory results from studies of animal infection models (*3*). A common belief is that if a clone from a patient with CA–*S. aureus* infection is positive for PVL, the *S. aureus* is probably a more dangerous clone and the patient would require specific care (*4*).

In the United States, virtually all CA-MRSA infections are caused by a PVL-positive clone of pulsed-field type USA300 (*5*). CA-MRSA infections with USA300 have also occasionally occurred outside the United States and adjacent regions. However, according to a recent study, they are derived from multiple importation events, suggesting that further spread in those locations is unlikely (*6*). Rather, global CA-MRSA infections are caused by geographically divergent clones that are unrelated to USA300. Like USA300, most of them contain PVL genes (*2*), although PVL is extremely rare in hospital-associated MRSA clones. This epidemiologic correlation is the predominant basis of the notion that PVL is causally associated with the enhanced virulence potential of CA-MRSA clones (*7*). Of note, despite generally enhanced virulence in animal models at levels similar to that of USA300 (*8*), the number of infections caused by global CA-MRSA clones remains limited (*9*,*10*). This situation raises the question of whether non-USA300 global CA-MRSA lineages have the potential to further intensify infection frequency and severity, and if so, whether PVL would be a necessary factor in such a scenario.

The CA-MRSA lineage that predominates in China and many other parts of Asia, thus threatening the largest global population, is sequence type (ST) 59 (*10*). Recent studies performed in Taiwan and northern Vietnam found a correlation between a PVL-positive subset of ST59 (Taiwan clone) and infection, but PVL-negative ST59 (Asia–Pacific clone) was found to be a largely noninfectious colonizer (*11*,*12*). Therefore, a causal relationship between PVL and infection has also been proposed for that CA-MRSA lineage.

## The Study

We studied *S. aureus* isolates collected over 10 years (2005–2014) at Shanghai Renji Hospital, Shanghai, China, a large teaching hospital at which ≈10,000 patients from the entire Shanghai metropolitan area are admitted each day. We obtained 2,048 infectious *S. aureus* isolates and characterized them by multilocus sequence and *spa* typing, antibiotic resistance profiling, determination of the staphylococcal cassette chromosome (SCC) *mec* type (encoding methicillin resistance), and analytical PCR to determine presence of the *lukSF* genes encoding PVL. For isolates obtained during 2005–2010, we investigated randomly selected subsets (100 isolates/year); for isolates obtained during 2011, 2012, and 2014, we investigated all isolates. No isolates were collected in 2013. CA–*S. aureus* was defined as an isolate obtained from either an outpatient or an inpatient (including from general and urgent care and emergency rooms) <24 h after hospital admission, who lacked risk factors (contact with the hospital environment in the 6 months preceding the culture, *S. aureus* infection history, residence in a long-term care facility in the 12 months before culture, presence of a central vascular catheter at the time of infection, or recent use of antimicrobial drugs). These data were obtained by a review of medical records. The study was approved by the ethics committee of Renji Hospital, School of Medicine, Shanghai Jiaotong University, Shanghai (protocol RJ-H-2015–0221).

The percentage of methicillin resistance in the *S. aureus* infectious isolates was high, as is generally reported for China (*13*), and remained stable (at ≈70%) over the past 10 years (Table; Figure, panel A). In contrast, during the same time, resistance among CA–*S. aureus* infections rose considerably, from 21% to 43% (p = 0.0108, Fisher exact test). While the percentage of HA-MRSA infections slightly decreased and that of CA–methicillin-sensitive *S. aureus* infections remained stable, CA-MRSA infections increased significantly, from 2.4% (12/500 total *S. aureus* infections) during 2005–2010 to 7.4% (35/470) in 2014 (p = 0.0003, Fisher exact test) (Table; Figure, panel A). ST59 dominated among CA-MRSA infections; increasing relative frequency reached a level of 71.4% in 2014 (Table; Figure, panel A). This finding links the observed surge in the CA-MRSA infection rate nearly exclusively to the ST59 lineage.

**Table:** Characteristics of *Staphylococcus aureus* isolated at Shanghai Renji Hospital, Shangai, China, 2005–2014*

Year	Total†	No. (%)											
		HA-MSSA	HA-MRSA	CA-MSSA	CA-MRSA	Invasive among CA-MRSA	CA	HA	MSSA	MRSA	ST59	ST59 CA-MRSA	Invasive among ST59 CA-MRSA
2005–2010	500	105 (21)	340 (68)	43 (8.6)	12 (2.4)	5 (42)	55 (11)	445 (89)	148 (30)	352 (70)	15 (3.0)	4 (0.8)	2 (50)
2011	600	124 (21)	398 (66)	57 (9.5)	21 (3.5)	14 (67)	78 (13)	522 (87)	181 (30)	419 (70)	24 (4.0)	9 (1.5)	6 (67)
2012	478	126 (26)	287 (60)	38 (7.9)	27 (5.6)	18 (67)	65 (14)	413 (86)	164 (34)	314 (66)	39 (8.2)	18 (3.8)	14 (78)
2014	470	114 (24)	275 (59)	46 (9.8)	35 (7.4)	18 (51)	81 (17)	389 (83)	160 (34)	310 (66)	45 (9.6)	25 (5.3)	13 (52)

*CA, community-associated; HA, hospital-associated; MRSA, methicillin-resistant *S. aureus*; MSSA, methicillin-sensitive *S. aureus*; ST, sequence type. †For 2005–2010, of a total of 2,681 isolates collected, 100 randomly selected isolates/year selected by using a random sample of data (SPSS, Chicago, IL, USA). For 2011, 2012, and 2014, all isolates obtained at the hospital were tested.

**Figure:**
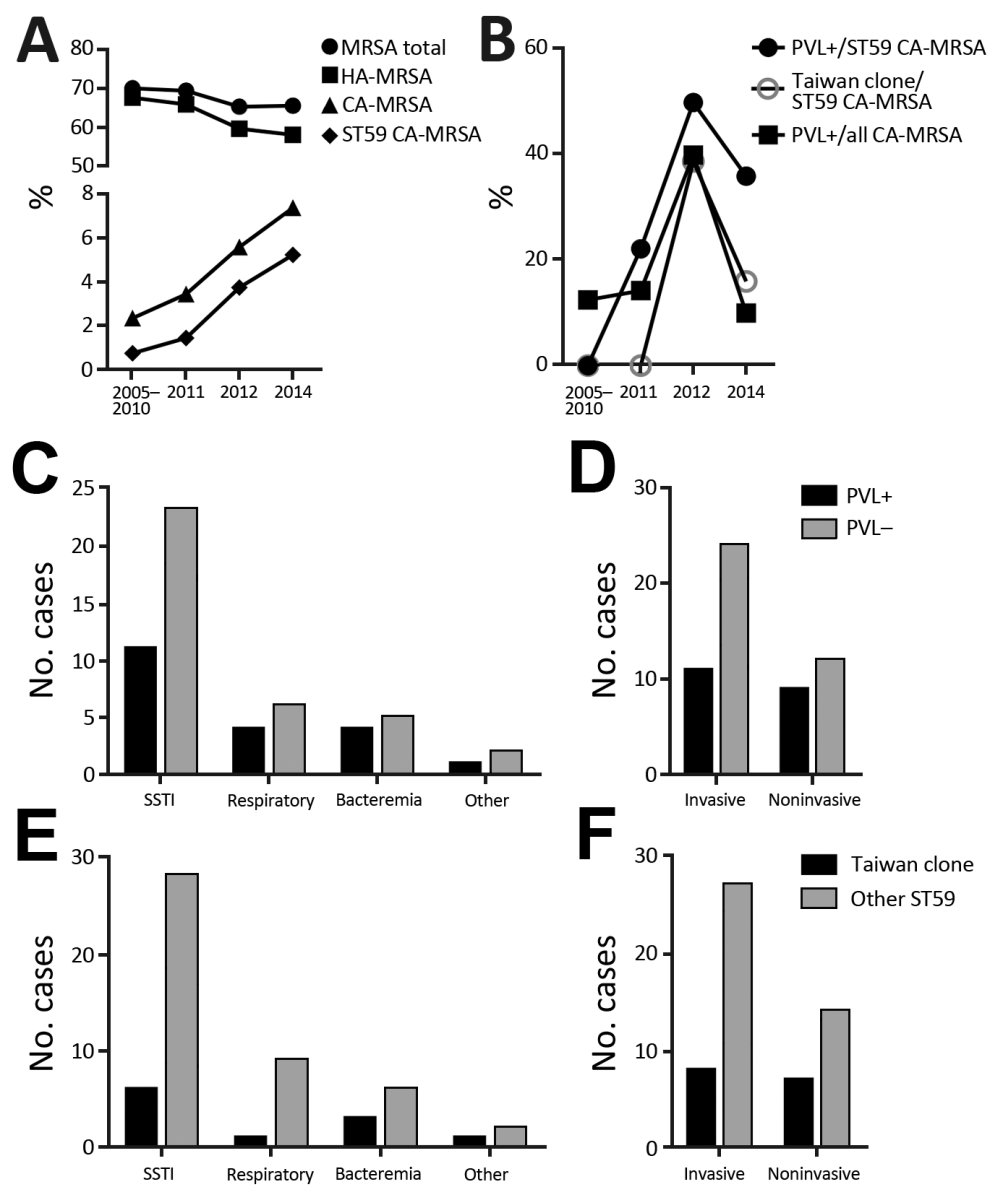
Epidemiology of MRSA in Shanghai, 2005–2014. Of infectious *Staphylococcus aureus* isolates obtained during 2005–2010, a random selection of 100 from each year were analyzed; of those obtained during 2001, 2012, and 2014, all isolates were analyzed. A) Percentages of MRSA (methicillin-resistant *S. aureus*) isolates among all obtained *S. aureus* isolates. B) Percentages of Panton-Valentine leukocidin (PVL)–positive clones among all or sequence type (ST) 59 community-associated (CA)–MRSA and of the Taiwan clone among ST59 CA-MRSA. C) Infection types from which ST59 CA-MRSA clones were obtained, differentiated by presence of PVL genes. D) Invasiveness of infections, differentiated by presence of PVL genes. E) Infection types from which ST59 CA-MRSA clones were obtained, differentiated by Taiwan clone versus other ST59 types. F) Invasiveness of infections, differentiated by Taiwan clone versus other ST59 types. HA, hospital acquired; SSTI, skin and soft tissue infection.

Of the 56 ST59 CA-MRSA infections, most (34 [61%]) were skin and soft tissue infections (i.e., spontaneous pyogenic skin abscesses), but a considerable number were respiratory (10 [18%]) or blood (9 [16%]) infections. The percentage of invasive infections (as defined by isolation from an otherwise sterile site, such as primary skin and soft tissue infection, with subsequent isolation from the blood, lung, or other otherwise sterile body fluids) among ST59 CA-MRSA patients was high at 62.5% (35/56). Fatality rate was 14% (5/35) among patients with invasive infections. Multidrug resistance was frequent. In addition to being resistant to β-lactams, most of the recently (2014) isolated ST59 CA-MRSA was also resistant to erythromycin (23/25; 92%), clindamycin (23/25; 92%), gentamicin (2/25; 8%), levofloxacin (3/25; 12%), trimethoprim/sulfamethoxazole (4/25; 16%), fosfomycin (3/25; 12%), or rifampin (5/25; 20%). None was resistant to tetracycline, linezolid, or vancomycin.

The ST59 CA-MRSA isolates were genetically heterogeneous and belonged to 7 *spa* types, predominantly t437 (31/56; 55%), t216 (12/56; 21%), and t441 (6/56; 11%). This finding is in contrast to the scenario described for USA300 CA-MRSA isolates, which are closely related (*14*), and suggests independent acquisition of SCC*mec* elements by genetically divergent parental ST59 methicillin-sensitive *S. aureus* strains.

Of note, only 20 (36%) of the 56 ST59 CA-MRSA isolates that we obtained contained the *lukSF* genes encoding PVL (Figure, panel B), and presence of the PVL genes was not correlated with more severe (i.e., invasive) infection (Figure, panel C). The PVL-positive Taiwan clone (*spa* types t437/t441, *lukSF*^+^, SCC*mec* V) was responsible for only 20% of cases (Figurepanel D). Moreover, while the percentage of PVL-positive ST59 CA-MRSA isolates and those belonging to the Taiwan clone increased in 2012, probably because of dissemination of the Taiwan clone into China, those numbers recently declined, indicating that PVL and the Taiwan clone are not main driving forces explaining the increase and current high percentage of CA-MRSA infections in Shanghai (Figure, panel B). Also, these subsets were not correlated with a specific infection type (Figure, panels C,D). Last, the Taiwan clone was not more frequently involved with invasive infections than were other ST59 CA-MRSA isolates (Figure, panel D).

## Conclusions

CA-MRSA infections caused by a non-USA300 clone increased significantly in a highly populated area in China. Whether our findings are representative of all of China and adjacent countries remains to be addressed. Our findings do not support the previously indicated correlation of the PVL-positive ST59 subset (Taiwan clone) with infection (*11*,*12*). Thus, our study provides epidemiologic evidence challenging the widespread notion about a significant role of PVL in CA-MRSA dissemination in the ST59 lineage and in general. Inasmuch as our findings underscore the idea that the development of CA-MRSA clones is less limited to specific genetic backgrounds than previously thought, they underscore that novel successful CA-MRSA clones will probably continue to emerge.
